# Ichthyological ethnoknowledge of the “piabeiros” from the Amazon region, Brazil

**DOI:** 10.1186/s13002-021-00468-7

**Published:** 2021-06-29

**Authors:** Daniel da Silva Ladislau, Maiko Willas Soares Ribeiro, Philip Dalbert da Silva Castro, Jackson Pantoja-Lima, Paulo Henrique Rocha Aride, Adriano Teixeira de Oliveira

**Affiliations:** 1 Programa de Pós-Graduação em Recursos Pesqueiros e Engenharia de Pesca, Universidade Estadual do Oeste do Paraná, Toledo, PR 85903-000 Brazil; 2 Programa de Pós-graduação em Ciência Animal e Recursos Pesqueiros, Universidade Federal do Amazonas, Manaus, AM 69077-000 Brazil; 3 Instituto de Ciências Biológicas, Universidade Federal do Amazonas, Manaus, AM 69077-000 Brazil; 4 Instituto Federal de Educação, Ciência e Tecnologia do Amazonas, Presidente Figueiredo, AM 69735-000 Brazil; 5 Instituto Federal de Educação, Ciência e Tecnologia do Amazonas, Manaus, AM 69020-120 Brazil

**Keywords:** Ornamental fish, Artisanal fishers, Ethnoichthyology, Amazon

## Abstract

**Background:**

The capture of ornamental fish is one of the main economic activities of riverine families in the Amazon. However, studies regarding the local ecological knowledge of workers in this activity are still incipient. In view of this, we have studied and explored the local ecological knowledge of artisanal fishers who specialize in the capture of fish for the aquarium trade in the middle part of the Negro River basin and investigated issues related to the ecological aspects of the fish species that are targeted by this trade in the region.

**Methods:**

Therefore, we conducted semi-structured interviews and applied questionnaires to artisanal fishers of ornamental fish (*N* = 89), from the municipality of Barcelos, from January to April 2016.

**Results:**

In total, 41 popular names were cited, which correspond to four ethnocategories and 10 families. The main species were *Paracheirodon axelrodi* (12.5%), *Hemigrammus bleheri* (8.3%), *Ancistrus dolichopterus* (6.4%), *Symphysodon discus* (5.3%), and *Potamotrygon motoro* (3.8%). According to the fishers, the species of fish known in the region as “piabas” have a preference for living in clusters (28.9%) and carry out migratory movements (26.1%). The diet of local fish species reported by fisheries is diverse, though mainly based on periphyton (42.2%), and the reproductive cycle directly influenced by the period of flooding of rivers in the region (37.6%)

**Conclusion:**

Our study revealed that the fishers possess information on the ecological aspects of local ornamental fish species, many of which are consistent with scientific literature. The information presented may assist in the decision-making process for the management of local fishery resources and contribute to the resumption of growth and sustainability in the capture of ornamental fish.

## Introduction

Ethnoichthyology is an ethnoscience (a branch of ethnozoology) that aims to study the knowledge, use, and meaning of ichthyofauna for different human populations, in regard to behavioral and cognitive aspects [1]. In Brazil, ethnoichthyology is a field that has only recently started to be explored when compared to other countries; however, this and other ethnosciences have grown significantly in the country since the last decade, as demonstrated by the increase in publications and studies, which contribute to the consolidation of ethnobiology in Latin America [2–5].

Ethnoichthyological studies have contributed via new information on the biological and ecological aspects of the target species of tropical fisheries. This has been beneficial since data on fishing in coastal and continental areas are often not reliable, and the lack of information hinders the process of decision-making in management plans [6]. Thus, the ecological knowledge of fishers from various regions of the country has been used in fisheries management, due to the fact that these actors have a detailed knowledge of the ecological, behavioral and classificatory aspects of fish [5–7].

In the Amazon, the capture of ornamental fish is responsible for the livelihood of a number of fishing communities, and occurs in the main tributaries of the Xingu [8, 9], Tapajós [10], Solimões [11], Purus [12] and Negro Rivers [13–20]. In the middle of the Negro River region, this activity is artisanal, selective and practiced by local fishers who are popularly known in the region as “piabeiros”. These fishers, through years of experience, have acquired a deep knowledge about local natural resources [15, 19].

In the Amazon Rainforest there are only two seasons: wet and dry, and the start of the dry season is when most of the fishing for piaba takes place [21]. For the fish that are captured, it is somewhat similar to a rescue operation; for example, a cardinal tetra (*Paracheirodon axeroldi* (Schultz, 1956)), one of the most popular piaba species, would be lucky to survive a year in the wild [21]. In a home aquarium, a cardinal tetra might live to two, three, or more years. In this sense, it may be considered to be the world’s most benign fishery [21]. Chao et al. [22] affirm that the trade in ornamental fish in the Amazon is fundamental to the maintenance of the forest. The authors created the slogan “buy a fish, save a tree,” which implies that the ornamental fish trade provides income for the riverine population and thus avoids the need to cut down trees as a means of income.

Despite the recognition of the importance of the capture of ornamental fish in the Amazon region, there are still few studies that focus on local ecological knowledge of artisanal fishers in the region [9, 12, 17]. In addition, the Negro River has a rich and diversified ichthyofauna with approximately 1,165 species. Many of these species are endemic to this basin and have not yet been cataloged or described, and some are captured and traded for the international aquarium market [23]. The traditional knowledge of riverine communities regarding the ecological aspects of species is often ignored by fisheries resource managers, thus causing relevant information to be lost.

In view of this, we have studied and explored the local ecological knowledge of artisanal fishers who specialize in the capture of fish for the aquarium trade in the middle part of the Negro River basin, and investigated issues related to the ecological aspects of the fish species that are targeted by this trade in the region.

## Materials and methods

### Study area

**Fig. 1:**
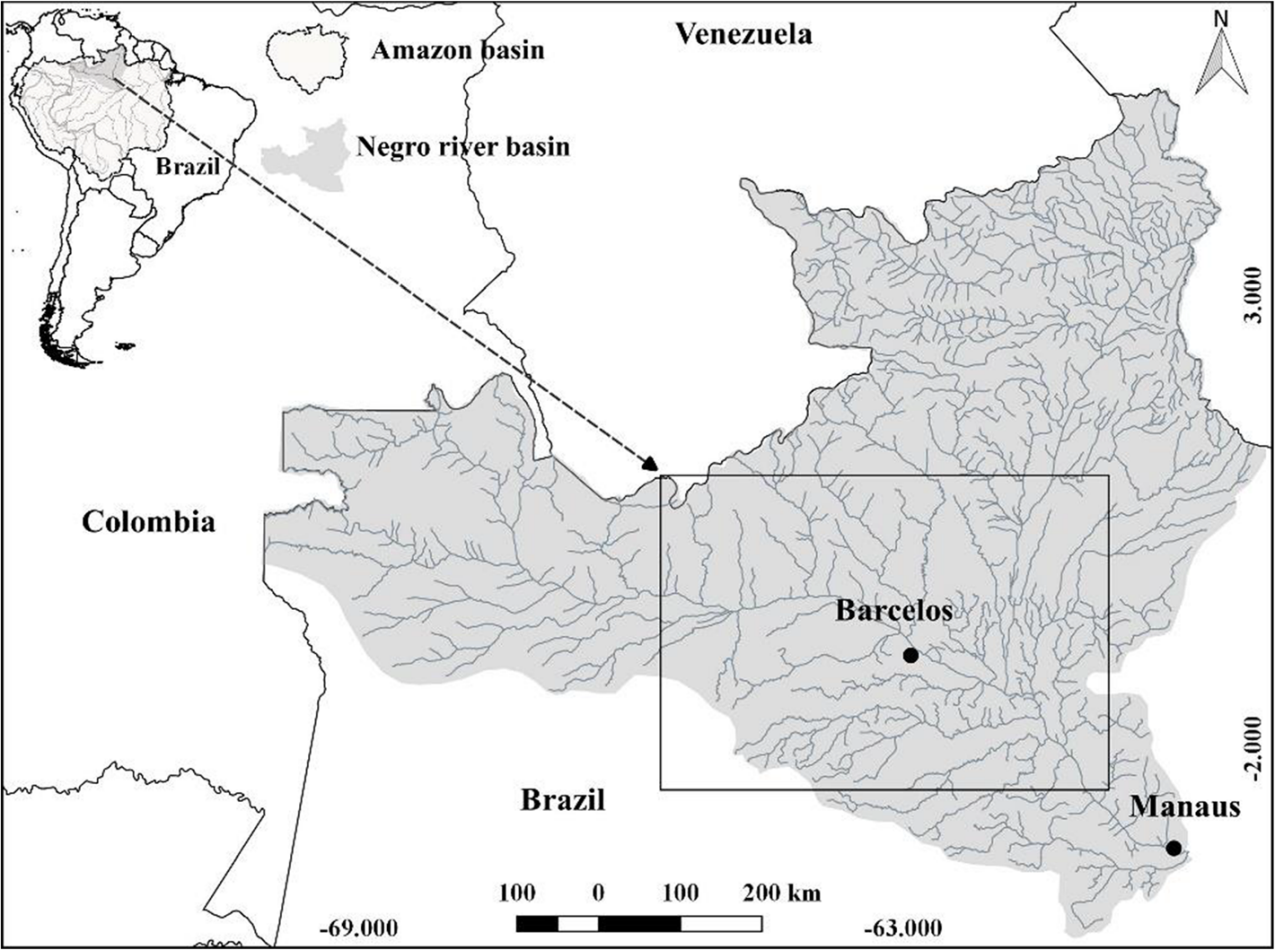
Region of the municipality of Barcelos located in the Negro River basin, Amazonas state, Brazil

The present study was carried out in the urban and riverine areas of the municipality of Barcelos (Fig. 1). Barcelos was the first capital of the state of Amazonas from 1758 until 1808. It is located on the right bank of the Negro River, 496 km from the capital Manaus by river and, in regard to territorial extension, it is considered the largest municipality in the Amazonas state with 112,450,769 km^2^, and has a population of 25,589 thousand inhabitants [24].

The exploration of ornamental fish in Barcelos started with Herbet Axelrod in 1955, when he visited the region looking for discus fish, probably the *Symphysodon discus* (Heckel, 1840), but ended up discovering the cardinal tetra *P. axeroldi* [22]. Within a year, the export business was flourishing, and a fishing operation was set up in Barcelos, which employed 50 workers [22]. The connection with the Negro River and the cardinal tetra continues to this day though, and ensures the preservation of the piaba and its habitat, as well the well-being of the local fishers [22].

### Interviews

All stages of this study received authorization and followed the protocols involving human beings established by the Research Ethics Committee (CEP) and was registered on “Plataforma Brasil” (N^o^.53847316.6.0000.5015 and 2.238.505). Semi-structured interviews were conducted with the application of questionnaires to artisanal ornamental fishers (*N* = 89) in the urban and riverine areas of the municipality of Barcelos, in the period from January to April, 2016 (Table 1).

**Table 1:** Interview locations in the municipality of Barcelos, middle Negro River basin, Amazonas

Interview locations	Fishers interviewed (n)	Percentage (%)
City of Barcelos	52	58.4
Ponta da Terra	8	8.9
Santa Inês	4	4.4
Daracuá	8	8.9
Mulufú	6	6.7
Romão	3	3.3
Elesbão	4	4.4
Bacabal	2	2.2
Jaqueira	2	2.2
**Total**	89	100

**Fig. 2:**
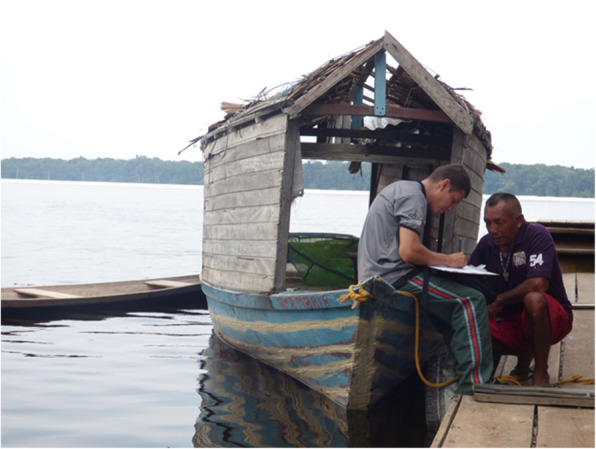
Interview with the piabeiros

The participants in this research were randomly selected in the city of Barcelos and in the riverine communities; however, in this study we considered only the information from artisanal fishers who identified themselves as “piabeiros” (Fig. 2). The interviewees were asked questions related to the ecological aspects (behavior, diet, reproduction) of the species of fish targeted in the ornamental fish trade in the region, such as, a) Which fish species do you fish for? (a) Do you catch fish for the local aquarium trade? b) Where do the fish live? c) What do the fish eat? and d) When is the spawning season of the fish? The questions followed the same order for all respondents.

### Data analysis

The data obtained in the interviews were tabulated in spreadsheets and analyzed using descriptive statistics based on the frequency of responses from the fishers. The words and expressions of the fishers were kept in their original form to guarantee the accuracy of the information.

For identification and taxonomic classification of fish species, we compared the descriptions made by the fishers in the interviews with the information contained in the scientific literature and with the FishBase database. Consultations were also held with specialist fishers (experienced in the practice of ornamental fishing) and local fish traders, in order to obtain information related to the ornamental species traded in the region.

Due to the amount and diversity of information cited by fishers during the interviews, we chose to group information into categories for better presentation and discussion. In addition, fishers cited more than one piece of information in a single category (e.g., types of habitats and food aspects), and in this case we chose to record the relative frequency of information for both categories (Table 2).

**Table 2:** Categories and variables created based on local ecological knowledge of fishers

Categories	Data variables
Behavioral aspects	Lives in shoals (species of fish that usually live in the water column in groups with individuals of the same or another species), solitary (species of fish that do not live in aggregate form), migratory (species of fish that perform migratory movements) and resident (species of fish generally demersal, considered not making migratory movements)
Habitat types	River, lake, beach (sand banks formed in the period when river levels are lowest in the Amazon), stream, flooded forest (igapó), floodplain, aquatic vegetation, river bank
Food aspects	Periphyton (slime), fish, plant material (fruits, flowers, leaves, vegetable debris and grass), worm (Oligochaeta), detritus (sand, mud, clay and water), insects (spiders and mosquitoes larvae (Diptera)), non-plant prganic material (decaying animal remains, blood, eggs from other fish), shrimp (Decapoda), crab (Decapoda), snails (Gastropoda)
Breeding season of species	Rising (April to June), full (July to August), ebb (September to November) and dry (December to March)

## Results

### Ethnoichthyology of fishers of ornamental fish

**Fig. 3:**
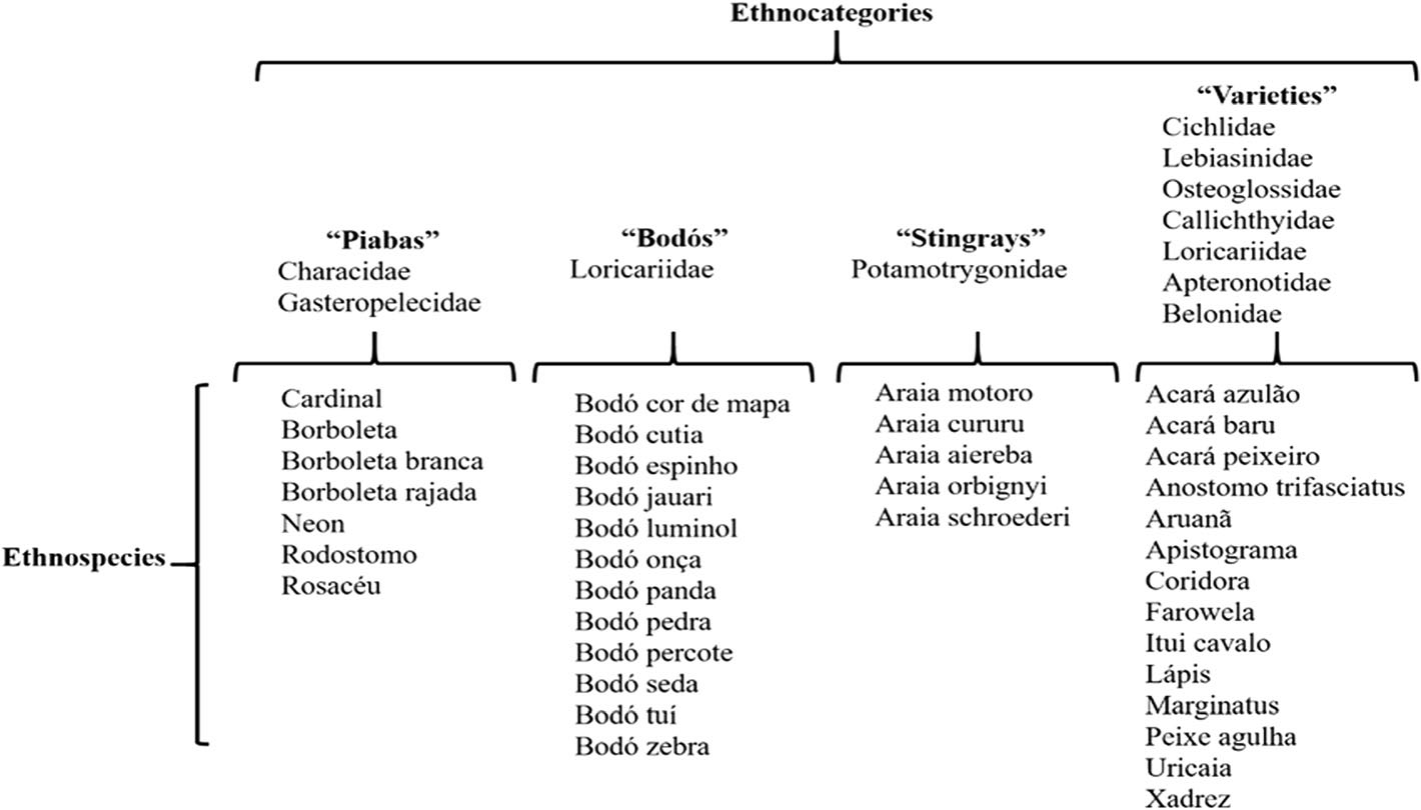
Ethnocategories of ornamental fish species according to fishers

In all, the interviewed fishers cited 41 ethnospecies (popular names) when referring to the species of fish caught and traded in the region for ornamental purposes (Table 3). The categories mentioned corresponded to 10 families and 4 ethnocategories, a group in which several ethnospecies of the same family or genus are aggregated; for example, the group “Araias” is formed by fish of the Potamotrygonidae family (Fig. 3).

**Table 3:** List of ethnospecies cited in interviews by fishers (“piabeiros”). (*n* = number of citations by interviewees)

Family	Local name/scientific name	Relative frequency (%)	Absolute frequency (n)
Cichlidae	Acará disco *Symphysodon discus* Heckel, 1840	5.38	34
	Acará azulão^a^	0.16	1
Cichlidae	Acará baru *Uaru amphiacanthoides* Heckel, 1840	0.16	1
Cichlidae	Acará peixeiro^a^	0.16	1
Lebiasinidae	Anostomo trifasciatus *Nannostomus trifasciatus* Steindachner, 1876	0.32	2
Cichlidae	Apistograma *Apistogramma* spp	3.96	25
Potamotrygonidae	Araia aireba *Paratrygon aiereba* Müller and Henle, 1841	0.95	6
Potamotrygonidae	Araia cururu *Potamotrygon wallacei* Carvalho, Rosa and Araújo, 2016	3.64	23
Potamotrygonidae	Araia motoro *Potamotrygon motoro* Müller and Henle, 1841	3.80	24
Potamotrygonidae	Araia orbignyi *Potamotrygon orbignyi* Castelnau, 1855	0.16	1
Potamotrygonidae	Araia Schroederi *Potamotrygon schroederi* Fernández-Yépez, 1958	0.16	1
Potamotrygonidae	Araia *Potamotrygon* spp	4.91	31
Osteoglossidae	Aruanã *Osteoglossum* spp	0.32	2
Loricariidae	Bodó cor de mapa *Peckoltia* spp	0.16	1
	Bodó cutia^a^	0.63	4
Loricariidae	Bodó espinho *Pseudocanthicus* spp	0.79	5
Loricariidae	Bodó jauari *Loricarridae* spp	0.95	6
Loricariidae	Bodó luminol *Ancistrus* spp	0.32	2
Loricariidae	Bodó onça *Pterygoplichthys gibbiceps* Kner, 1854	3.80	24
	Bodó panda^a^	0.16	1
	Bodó pedra^a^	0.16	1
Loricariidae	Bodó percote *Peckoltia* spp	1.11	7
Loricariidae	Bodó seda *Ancistrus dolichopterus* Kner, 1854	6.49	41
Loricariidae	Bodó^a^	1.42	9
	Bodó tuí^a^	0.16	1
Loricariidae	Bodó zebra *Peckoltia* spp	0.47	3
Gasteropelecidae	Borboleta *Carnegiella* spp	9.18	58
Gasteropelecidae	Borboleta branca *Carnegiella marthae* Myers, 1927	0.16	1
Gasteropelecidae	Borboleta rajada *Carnegiella strigata* Günther, 1864	0.16	1
Characidae	Cardinal *Paracheirodon axelrodi* Schultz, 1956	12.50	79
Callichthyidae	Coridora *Corydoras* spp	0.79	5
Loricariidae	Farowela *Farlowella* spp	0.16	1
Apteronotidae	Ituí cavalo *Apteronotus albifrons* Linnaeus, 1766	0.16	1
Lebiasinidae	Lápis *Nannostomus* spp	6.65	42
Lebiasinidae	Marginatus *Nannostomus marginatus* Eigenmann, 1909	2.53	16
Characidae	Neon *Paracheirodon innesi* Myers, 1936	3.64	23
Belonidae	Peixe agulha *Potamorrhaphis guianensis* Jardine, 1843	0.32	2
Characidae	Rodostomo *Hemigrammus bleheri* Géry and Mahnert, 1986	8.39	53
Characidae	Rosacéu *Hyphessobrycon* spp	7.75	49
	Uricaia^a^	0.16	1
Cichlidae	Xadrez *Dicrossus* spp	6.80	43
Total		100	632

^a^Species for which it was not possible to carry out the identification based on the description of the interviewees

**Fig. 4:**
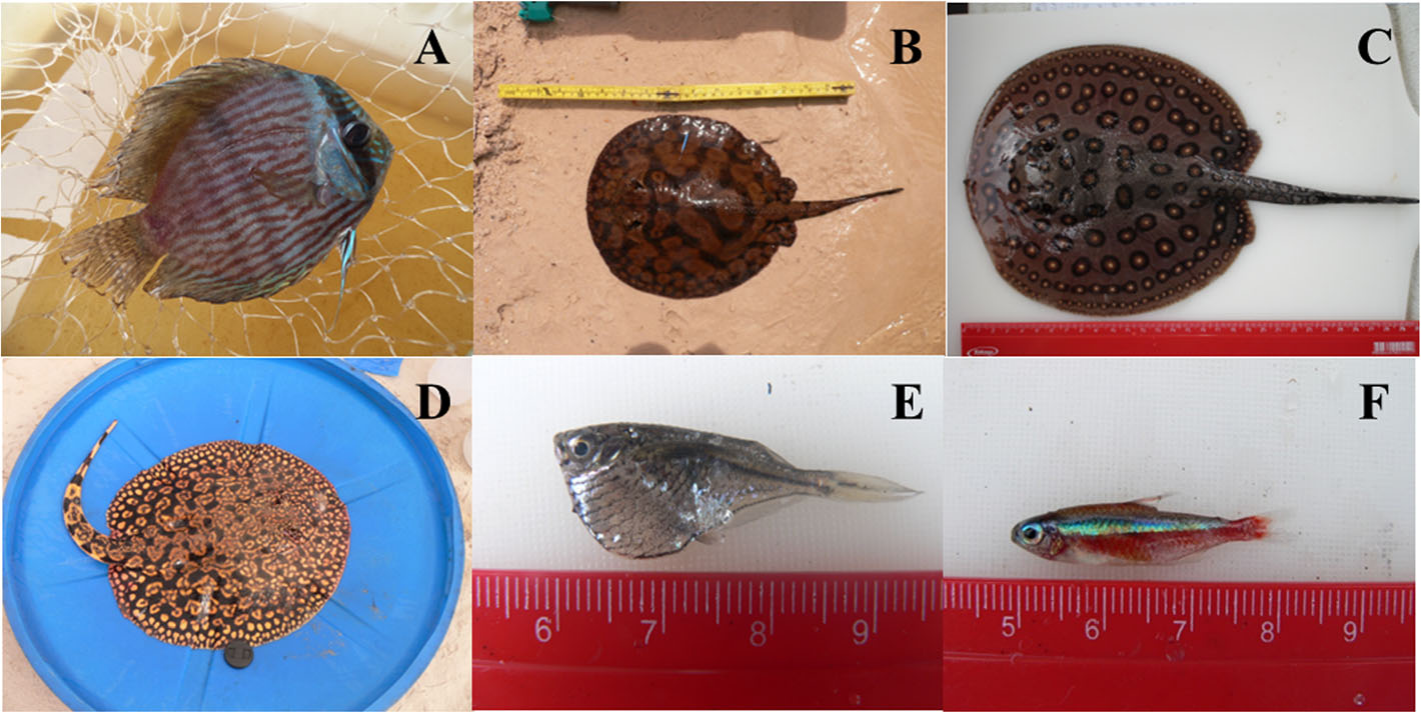
Some species of ornamental fish reported by piabeiros. (**A**) Acara disco—*Symphysodon discus*. (**B**) Araia cururu—*Potamotrygon wallacei*. (**C**) Araia motoro—*Potamotrygon motor*o. (**D**) Araia schroederi—*Potamotrygon schroederi*. (**E**) Borboleta branca—*Carnegiella marthae.* (**F**) Cardinal—*Paracheirodon axelrodi*

The Loricariidae family showed a predominance of ten ethnospecies, especially the bodó seda—*Ancistrus dolichopterus* (kner, 1854) (6.4%) and bodó onça—*Pterygoplichthys gibbiceps* (Kner, 1854) (3.8%). Fishers also mention five species of stingray (Potamotrygonidae), namely araia motoro—*Potamotrygon motoro* (Müller and Henle, 1841) (3.8%), araia cururu—*Potamotrygon wallacei* (Carvalho, Rosa and Araújo, 2016) (3.6%), araia aiereba—*Paratrygon aiereba* (Müller and Henle, 1841) (0.95%), araia schroederi—*Potamotrygon schroederi* (Fernández-Yépez, 1958) (0.16%), and araia orbignyi—*Potamotrygon orbignyi* (Castelnau, 1855) (0.16%). The main ethnospecies mentioned were cardinal—*Paracheirodon axelrodi* (Schultz, 1956) (12.5%), rodostomo—*Hemigrammus bleheri* (Géry and Mahnert, 1986) (8.3%), bodó seda—*Ancistrus dolichopterus* (6.4%), acará disco—*Symphysodon discus* (5.3%), and araia motoro—*Potamotrygon motoro* (3.8%); together, these represent 36.5% of all ethnospecies cited by fishers (Table 3) (Fig. 4).

Some ethnospecies were reported only once, such as acará azulão, acará baru, acará peixeiro, araia orbignyi, araia schroederi, bodó cor de mapa, bodó panda, bodó pedra, bodó tuí, borboleta branca, borboleta rajada, farowela, ituí cavalo, and uricaia (Table 3).

We observed that the fishers in the region create clusters of ethnospecies, which we call ethnocategories, called “piabas,” bodós, araias, and varieties (a group which includes several ethnospecies from different families and genres) (Fig. 2).

### Behavioral aspects of ornamental fish

According to fishers, the majority of ornamental fish species called “piabas” prefer to live in groups (28.9%) and make migratory movements (26.1%) known locally as “arribação,” which is an event that consists of the displacement of some species in the seasonal period of the rise or ebb of the waters of the region’s rivers, and includes species such as cardinal (*P. axelrodi*), borboleta (*Carnegiella* spp.), rodostomo (*H. bleheri*) and rosacéu (*Hyphessobrycon* spp.) (Table 4).

**Table 4:** Behavior of ornamental fish according to the fishers (“piabeiros”) of Barcelos, Amazonas

Species	Solitary		Lives in shoals		Resident		Migratory		No answer
	%	n	%	N	%	N	%	n	
Acará disco	0.68	1	6.62	21	2.82	5	5.94	17	12
Acará azulão	0.68	1			0.56	1			
Acará baru	0.68	1			0.56	1			
Acará peixeiro	0.68	1			0.56	1			
Anostomo trifaciato			0.32	1			0.35	1	1
Apistograma	0.68	1	2.21	7	1.69	3	1.75	5	17
Araia aireba	2.74	4	0.63	2	2.82	5	0.35	1	
Araia cururu	13.01	19	0.95	3	9.60	17	1.75	5	1
Araia motoro	13.01	19	0.95	3	11.30	20	0.70	2	2
Araia orbignyi			0.32	1			0.35	1	
Araia schroederi			0.32	1			0.35	1	
Araia	16.44	24	0.63	2	10.73	19	2.45	7	5
Aruanã			0.32	1			0.35	1	1
Bodó cor de mapa									1
Bodó cutia	0.68	1	0.32	1	0.56	1	0.35	1	2
Bodó espinho	2.05	3			1.69	3			2
Bodó jauari	2.74	4			2.26	4			2
Bodó luminol	0.68	1			0.56	1			1
Bodó onça	11.64	17	0.63	2	7.91	14	1.75	5	5
Bodó panda									1
Bodó pedra									1
Bodó percote	3.42	5			2.82	5			2
Bodó seda	17.12	25	2.84	9	15.25	27	2.45	7	7
Bodó	4.11	6	0.63	2	3.95	7	0.35	1	1
Bodó tui	0.68	1			0.56	1			
Bodó zebra	0.68	1					0,35	1	2
Borboleta	1.37	2	13.88	44	6.78	12	11.89	34	12
Borboleta branca			0.32	1			0.35	1	
Borboleta rajada			0.32	1			0.35	1	
Cardinal			23.03	73	1.69	3	24.48	70	6
Coridora			0.95	3	0.56	1	0.70	2	2
Farowela			0.32	1			0.35	1	
Itui cavalo									1
Lápis	0.68	1	5.99	19	2.82	5	5.24	15	22
Marginata			2.52	8			2.80	8	8
Neon			5.99	19	1.13	2	5.94	17	4
Peixe agulha									2
Rodostomo	2.74	4	11.36	36	4.52	8	11.19	32	13
Rosacéu	2.05	3	10.41	33	3.95	7	10.14	29	13
Uricaia			0.32	1			0.35	1	
Xadrez	0.68	1	6.94	22	2.26	4	6.64	19	20
Total	100	146	100	317	100	177	100	286	169

In relation to the behavioral aspects mentioned above, we can observe such examples of the behavior of the cardinal (*P. axelrodi*) according to what was reported by the interviewee, as reproduced below:

[…] They are always swimming in a shoal, the large ones all mixed in with the young (WPS, 34 years old).

Fishers reported that fish they considered being ”sedentary” choose to live alone (13.3%) and do not carry out migratory movements and were considered resident species (16.1%). The species cited by fishers with such characteristics were mainly araias (Potamotrygonidae), as cururu (*P. wallacei*) and motoro (*P. motoro*), in addition to the bodó onça (*P. gibbiceps*) and bodó seda (*A. dolichopterus*) (Table 4).

According to fishers, the ornamental fish in this region inhabit different types of environments, which change according to the seasonal regime of the local rivers. In this scenario, streams (34.1%), locally known as igarapés, were mentioned as the main habitat for ornamental fish species, followed by lakes (7.1%) and igapós (5.5%) which are a portion of forests that are flooded by the Negro River’s black waters. Aquatic vegetation and riverbanks were the environments that were considered to have the lowest occurrences of the species (1.4% and 0.15% respectively).

Fishers also reported that some environments, such as streams and the igapós, are inhabited by fish species belonging to the piabas (e.g., cardinal—*P. axelrodi*), rodostomo (*H. bleheri*), borboleta (*Carnegiella* spp), and rosacéo (*Hyphessobrycon* spp) and other varieties (e.g., acará disco—*S. discus*). For the bodó seda (*A. dolichopterus*) species, the igarapé was mentioned as the main habitat; however, for the main species belonging to the ethnocategory of araias, the beach and lake environments were the most cited, while the igarapé and lake were related to fish habitats of the ethnocategory bodó (Table 5).

**Table 5:** Types of habitats of ornamental fish mentioned by artisanal fishers (“piabeiros”)

Species	Habitat types																No answer
	River		Lake		Stream		Beach		Forest igapó		Flood plain lake		Aquatic vegetation		River bank		
	%	N	%	N	%	n	%	n	%	n	%	n	%	n	%	n	
Acará disco	5	1	2.08	1	6.99	16			13.51	5							11
Acará azulão					0.44	1											
Acará baru					0.44	1											
Acará peixeiro					0.44	1											
Anostomo trifaciato			2.8	1													1
Apistograma	5	1	2.8	1	2.62	6			5.41	2							17
Araia areba			4.17	2			5.88	1									3
Araia cururu			6.25	3	2.62	6	17.65	3									12
Araia motoro			6.25	3	3.06	7	23.53	4									11
Araia orbignyi							5.88	1									
Araia schroederi							5.88	1									
Araia	15	3	2.08	1	9.17	21							10	1			8
Aruanã	5	1															1
Bodó cor de mapa					0.44	1											
Bodó cutia			4.17	2	0.87	2	5.88	1									1
Bodó espinho			2.08	1	0.87	2			2.70	1							1
Bodó jauari			2.08	1	1.31	3	5.88	1	2.70	1							2
Bodó luminol			2.08	1	0.44	1	5.88	1									1
Bodó onça			4.17	2	3.06	7			8.11	3							12
Bodó panda					0.44	1											
Bodó pedra																	1
Bodó percote			2.08	1	0.44	1	11.76	2	2.70	1					100	1	3
Bodó seda			4.17	2	6.55	15			18.92	7							17
Bodó					2.62	6											3
Bodó tui																	1
Bodó zebra			2.08	1	0.44	1											1
Borboleta	10	2	6.25	3	6.99	16	11.76	2	8.11	3	12.50	2					34
Borboleta branca			2.08	1													
Borboleta rajada			2.08	1													
Cardinal	15	3	16.70	8	17.03	39			8.11	3	25	4	20	2			26
Coridora			2.08	1	0.87	2											2
Farowela					0.44	1											
Itui cavalo																	1
Lápis	5	1	4.17	2	5.24	12			2.70	1	12.50	2					25
Marginata	10	2	2.08	1	1.75	4							10	1			10
Neon	5	1	2.08	1	4.37	10			8.11	3	6.25	1					9
Peixe agulha																	2
Rodostomo	10	2			7.86	18					25	4	60	6			25
Rosacéu	10	2	10.4	5	6.55	15			10.81	4	18.75	3					23
Uricaia					0.44	1											
Xadrez	5	1	4.17	2	5.24	12			8.11	3							28
Total	100	20	100	48	100	229	100	17	100	37	100	16	100	10	100	1	292

### Feeding habits of the ornamental fish

According to the reports of the fishers, the ornamental fish species in the region have diversified diets; however, slime or periphyton (42.2%) is the main component of the diet of most species of piabas, as well as the types of bodó and some of the other varieties (e.g., acará disco *S. discus* and lápis *Nannostomus* spp) (Table 6). In relation to fish with fish-based diets, the majority of fishers associated this habit with fish of the Potamotrygonidae family. However, during the interviews, there were some fishers who also associated the consumption of detritus and earthworms (Table 6) with the fish of this family:

[…] it (the stingray) likes mud and those old leaves from the bottom [...] eats everything, worms, shrimp, crab, fish (HD, 58 years old).

**Table 6:** Feeding habits of ornamental fish according to artisanal fishers (“piabeiros”)

Species	Ornamental fish food items																				No answer
	Shrimp Decapoda		Snails Gastropoda		Crab Decapoda		Insects		Worm Oligochaeta		Fish		Detritus		Periphyton		Plant material		Non-plant organic material		
	%	n	%	N	%	N	%	n	%	n	%	N	%	n	%	n	%	n	%	n	
Acará disco							4.76	1			2.27	2			8.22	30	3.08	2	5.88	1	4
Acará azulão															0.27	1	1.54	1			
Acará baru															0.27	1	1.54	1			
Acará peixeiro															0.27	1	1.54	1			
Anostomo trifaciato															0.55	2					
Apistograma									2	1	1.14	1			3.01	11	3.08	2	5.88	1	14
Araia aireba	22.22	2			20	1			6	3	2.27	2	7.69	2	0.27	1			5.88	1	1
Araia cururu	33.33	3			60	3			32	16	11.4	10	19.23	5	0.27	1	3.08	2	5.88	1	7
Araia motoro	22.22	2	100	1	20	1			32	16	11.4	10	3.85	1	0.27	1	1.54	1			8
Araia orbignyi											1.14	1									
Araia schroederi											1.14	1									
Araia	22.22	2							28	14	17	15	30.77	8	1.10	4					16
Aruanã											1.14	1									1
Bodó cor de mapa															0.27	1					
Bodó cutia													3.85	1	1.10	4					
Bodó espinho							4.76	1							1.10	4					
Bodó jauari													3.85	1	1.64	6					
Bodó luminol													3.85	1	0.55	2					
Bodó onça							4.76	1					7.69	2	5.48	20					4
Bodó panda															0.27	1					
Bodó pedra															0.27	1					
Bodó percote													3.85	1	1.92	7					
Bodó seda							4.76	1					7.69	2	10.14	37					4
Bodó															1.92	7					2
Bodó tui															0.27	1					
Bodó zebra															0.82	3					
Borboleta							38.10	8			2.27	2	3.85	1	8.49	31	13.85	9	5.88	1	27
Borboleta branca															0.27	1					
Borboleta rajada															0.27	1					
Cardinal							19.05	4			21.6	19			13.42	49	30.77	20	17.65	3	30
Coridora															1.10	4					1
Farowela																					1
Itui cavalo															0.27	1					
Lápis															5.48	20	4.62	3	5.88	1	22
Marginata							4.76	1			1.14	1			2.19	8	3.08	2			8
Neon											3.41	3			4.11	15	3.08	2			8
Peixe agulha															0.55	2					
Rodostomo							14.29	3			11.4	10			8.22	30	12.31	8	11.76	2	23
Rosacéu							4.76	1			9.09	8			8.77	32	9.23	6	29.41	5	17
Uricaia																					1
Xadrez											2.27	2	3.85	1	6.58	24	7.69	5	5.88	1	19
Total	100	9	100	1	100	5	100	21	100	50	100	88	100	26	100	365	100	65	100	17	218

In some cases, fishers linked the consumption of fish to the diet of species such as cardinal (*P. axelrodi*), rodostomo (*H. bleheri*), and rosacéu (*Hyphessobrycon* spp.). Crustaceans, such as shrimp (1.04%) and crab (0.58%), followed by snails (0.12%), were less often mentioned (Table 6).

### Reproductive aspects of ornamental fish

For the fishers, the reproductive cycle of the species is directly influenced by the seasonal dynamics of the water levels of the rivers. The period when the river rises (37.6%) is the period that they considered to be the main breeding season for most species of ornamental fish in the region (Table 7), as demonstrated by the fishers’ knowledge reported below:

[…] Every ornamental fish has offspring in the flood and only migrates in the “arribação,” when they disappear (SCP, 45 years).

**Table 7:** Reproduction period of ornamental fish according to artisanal fishers (“piabeiros”)

Species	Reproductive seasons								No answer
	Rising		Full		Ebb		Low		
	%	n	%	N	%	n	%	n	
Acará disco	5.11	12	2.38	1	10.45	7	17.39	4	10
Acará azulão					1.49	1			
Acará baru					1.49	1			
Acará peixeiro					1.49	1			
Anostomo trifaciato									2
Apistograma	2.55	6	4.76	2	2.99	2			15
Araia aireba									6
Araia cururu	0.43	1			2.99	2			20
Araia motoro					2.99	2	4.35	1	21
Araira orbignyi									1
Araia schroederi									1
Araia	3.40	8	2.38	1	19.40	13	39.13	9	
Aruanã	0.43	1							1
Bodó cor de mapa									1
Bodó cutia	0.85	2							2
Bodó espinho	0.43	1			1.49	1			3
Bodó jauari					1.49	1			5
Bodó luminol									2
Bodó onça	2.13	5			4.48	3			8
Bodó panda									1
Bodó pedra									1
Bodó percote									7
Bodó seda	3.83	9	4.76	2	11.94	8			22
Bodó					2.99	2	26.09	6	1
Bodó tui									1
Bodó zebra	0.43	1							2
Borboleta	9.79	23	7.14	3	4.48	3	4.35	1	28
Borboleta branca									1
Borboleta rajada									1
Cardinal	23.83	56	35.71	15	4.48	3	4.35	1	4
Coridora	0.43	1			2.99	2			2
Farowela	0.43	1							
Itui cavalo									1
Lápis	6.38	15	7.14	3	4.48	3			21
Marginata	2.98	7	2.38	1	1.49	1			7
Neon	6.38	15	4.76	2	2.99	2			4
Peixe agulha									2
Rodostomo	10.21	24	14.29	6	4.48	3	4.35	1	19
Rosacéu	11.91	28	7.14	3	4.48	3			15
Uricaia	0.43	1							
Xadrez	7.66	18	7.14	3	4.48	3			19
Total	100	235	100	42	100	67	100	23	257

The fishers attributed the periods of ebb and when the river is full as periods of reproduction of the species of the ethnocategory araia (Potamotrygonidae) and bodó (Loricariidae) (Table 7). Some fishers demonstrated that they were unaware of the reproductive aspects of some species of local ornamental fish, as shown in excerpts from the following interviews:

[…] For the cardinal, we only find them with eggs (mature oocytes) at the time of the migratory season (species migration period). I think it has no male, because we never saw a cardinal in that period that did not have eggs (JNG, 46 years old).

[…] The cardinal spawns the young, do you know why? We only see the young - we do not see the eggs (ACL, 56 years old).

[…] You know, I've never seen this fish with young (RRS, 51 years old) (fisherman referring to the Borboleta (*Carnegiella* spp.).

## Discussion

The species and ornamental fish families described by the fishers in our study are among the most commercialized in the middle region of the Negro River basin [13, 15, 18, 19].

Like the cardinal, the fishers cited other ethnospecies that make up a large portion of the fish exported from the Amazon, such as rodostomo (*H. bleheri*), borboleta (*Carnegiella* spp.), acará disco (*S. discus*), and ethnocategories araias (Potamotrygonidae) and bodó (Loricariidae). Rodostomo ranks second on the list of the species most exported from the state, second only to the cardinal, and corresponds to about 6% of the total volume of fish exported (20 to 30 million fish exported annually from the state of Amazonas) [25].

According to [14], ornamental stingray fishing in the middle region of the Negro River basin is already a consolidated activity, and since the regulation of fishing and export of freshwater stingrays (quota system) came into force in 1998, approximately 130,000 rays have been exported from the region, the main species being *P. motoro*, *P. wallacei*, *P. schroederi*, *P. orbignyi*, *Potamotrygon leopoldi* (Castex and Castello, 1970), and *Potamotrygon henlei* (Castelnau, 1855).

Artisanal fishers from the middle Negro River basin demonstrated their knowledge of a vast diversity of species and presented their own way of classifying local fish through the formation of ethnocategories. In Brazil, several studies have sought to investigate the different ways of classifying fish caught by artisanal fishers, demonstrating that fishers make groupings of species in a hierarchical manner, based on morphological, behavioral, or ecological criteria [6, 16].

Some fish species in Table 3 depicted the absolute frequency of only one. This low representation is due to the enormous diversity that occurs in the locality, as well as the difficulty that the fisher has in classifying some species that were portrayed in this study, due to the similarity that they share with other species.

According to Begossi et al. [26], riverine fishers from the Amazon usually seek to identify fish species by their similarities in terms of morphology, diet, habitat, or behavior, classifying them as “cousins” or “relatives.” However, in our study, it is something that needs to be investigated through future studies in which such aspects are more thoroughly investigated.

Based on the fishers’ reports, it can be seen that the behavior, diet, and reproduction of ornamental fish species are related to seasonal fluctuations of the rivers in the region. Studies show that the seasonal variations in the water level of the Negro River favor the emergence of new habitats, such as lakes, temporary beaches, flooded fields, and igapós (flooded forests), which serve as a shelter and breeding and feeding grounds for aquatic communities, and such changes end up directly influencing the composition of the ichthyofauna [27, 28]. In the study by [29], in the region of the Anavilhanas National Park (middle Negro River region), the authors demonstrate how the hydrological cycle influences the composition and structure of fish assemblages in the local lakes and igapós, and the important role of igapós in maintaining the diversity and abundance of ichthyofauna in black waters is also emphasized.

Fish species of the ethnocategory “piabas” in their majority were considered by fishers as species that present behavior of coexistence in groups, and in the period of the floods of the rivers, they carry out migratory movements locally known as “arribação” (migration). Such behavior of the species of this group described by the fishers is consistent with that reported in other studies carried out in the middle region of the Negro River basin [30]. Formations of aggregations by species of ornamental fish have also been described in other regions of the Amazon, as is the case of the discus (*S. aequifasciatus*) in the lower Solimões river and lower Purus river in the Amazon, where in the dry season (September to November), schools of fish are concentrated around submerged tree branches on the banks of lakes and rivers [12, 31].

The differences between the types of waters in the rivers of the Amazon basin may influence the distribution of freshwater stingrays of the Potamotrygonidae family and act as a hydrological filter for the dispersion of fish species in this group [32]. In the study developed by [33], the authors observed ecophysiological differentiations in the preferences of environments between the species of rays (*P. motoro*, *P. wallacei*, and *P. aiereba*) in the middle of the Negro River region. Artisanal fishers from Barcelos and the riverine communities of the Negro River report that the local freshwater stingrays have a preference for lake areas, beaches, and rivers, which are the same habitats cited by fishers in [17]. The information described in the aforementioned studies may help us to understand the fact that the fishers consider the fish belonging to this ethnocategory as residents, since the species have different distribution patterns.

The streams in the region known as “igarapés” were identified as the main places where the species of ornamental fish live. This is the case of the Acará disco *S. discus*, a species from the lower regions of the Negro, Trombetas, and Abacaxis Rivers, for which streams (igarapés) are the main habitat [34]. This environment is also seen as the main artisanal fishing ground for ornamental fish in the region [15, 19].

The flood period favors a greater availability of food and shelter for fish, since there is a greater availability of space, for example, the igapó in the forest that appears seasonally [28]. According to fishers, the diet of ornamental fish species is quite diverse; however, periphyton was identified as the main food item of most species. It was observed that the ornamental fish species from the middle Negro River basin that were cited by fishers tend to inhabit igarapés and igapó forests at different times and occupy these environments in search of food [25, 30]. According to [35], the food base for some of the fish in the igarapé is composed of small terrestrial invertebrates (ants and termites) when they fall into the water, as well as the mosquito larvae (Diptera) present at the bottom of these environments.

Regarding the diet of the species, fishers reported that fish in the Potamotrygonidae family have a diet based on fish, in addition to shrimp, crab, and snail. According to [36], who analyzed the stomach contents of four species of freshwater stingrays (*P. motoro*, *P. orbignyi*, *P. wallacei* and *P. aiereba*) from the middle of the Negro River basin, it was observed that the diet of these stingrays was basically composed of fish, crustaceans, and insects. However, there are differences in proportions, possibly due to the different types of uses of microhabitats and foraging substrates of these fish. Feeding behavior and morphological characteristics can also influence the composition of the diet of these animals [37].

Some fishers reported that fish of the ethnocategory piabas (cardinal, rodostomo, and rosacéu) feed on fish, while fish of the ethnocategory araias fed on debris (mud, decomposing sand, and leaves) and earthworms. In the first case, such placement may be related to the type of food offered to catch fish, where the caught fish are kept for a certain period in ponds built with screens on the banks of the rivers by the “piabeiros” and fed with cooked fish meat, as observed in the field. While the statement that araias feed on earthworms and debris may be linked to the fact that these animals are partly associated with the substrate of rivers, lakes, streams, and beaches, between sand and mud, and this may have led fishers to relate this behavior to the eating habits of these fish.

Some species of fish in the Amazon show spawning synchronism with changes in the hydrological cycle of rivers and annual rainfall regimes and perform migrations called “piracema” [38]. As well as their feeding behavior, in the fishers’ view, the reproductive cycle of ornamental fish species is also influenced by annual variations in water levels in the region’s rivers, with the period of rising river levels being the main breeding season for the species. In the Amazon region, several species of ornamental fish demonstrate synchronism of the reproductive cycle with the initial phase of increasing water levels [25, 30, 31].

## Conclusions

In summary, our study demonstrated that the fishers of ornamental fish in the middle region of the Negro River basin, who are known as “piabeiros,” possess important information related to the behavioral, dietary, and reproductive aspects of local ornamental fish species. Although our methods are based on descriptive analysis and this may limit our conclusions, we believe that our number of respondents poses a representative sample of fishers and their ecological knowledge on local species.

We hope that our study will contribute to the emergence of new studies aimed at understanding the local ecological knowledge of fishers of ornamental fish species in the Amazon, since the existing information on the topic is still incipient. The information presented may assist in the decision-making process for the management of local fishery resources and contribute to the resumption of growth and sustainability of the capture of ornamental fish.

## Data Availability

All data generated or analyzed during this study are included in this published article.
